# Delayed formation of zero-valent selenium nanoparticles by *Bacillus mycoides* SeITE01 as a consequence of selenite reduction under aerobic conditions

**DOI:** 10.1186/1475-2859-13-35

**Published:** 2014-03-07

**Authors:** Silvia Lampis, Emanuele Zonaro, Cristina Bertolini, Paolo Bernardi, Clive S Butler, Giovanni Vallini

**Affiliations:** 1 Department of Biotechnology, University of Verona, Strada le Grazie 15, Verona 37134, Italy; 2 Department of Neurological and Movement Sciences, Piazzale L. A. Scuro, Verona 10 - 37134, Italy; 3 Biosciences, College of Life and Environmental Sciences, University of Exeter, Stocker Road, Exeter EX4 4QD, UK

**Keywords:** Bacillithiol, *Bacillus mycoides* SeITE01, Extracellular precipitation, Intracellular deposition, Ostwald ripening mechanism, Redox regulation, Selenite reduction, TEM analysis, Xenobiotic detoxification, Zero-valent selenium nanoparticles

## Abstract

**Background:**

Selenite (SeO_3_^2−^) oxyanion shows severe toxicity to biota. Different bacterial strains exist that are capable of reducing SeO_3_^2−^ to non-toxic elemental selenium (Se^0^), with the formation of Se nanoparticles (SeNPs). These SeNPs might be exploited for technological applications due to their physico-chemical and biological characteristics. The present paper discusses the reduction of selenite to SeNPs by a strain of *Bacillus* sp., SeITE01, isolated from the rhizosphere of the Se-hyperaccumulator legume *Astragalus bisulcatus*.

**Results:**

Use of 16S rRNA and GyrB gene sequence analysis positioned SeITE01 phylogenetically close to *B. mycoides*. On agarized medium, this strain showed rhizoid growth whilst, in liquid cultures, it was capable of reducing 0.5 and 2.0 mM SeO_3_^2−^ within 12 and 24 hours, respectively. The resultant Se^0^ aggregated to form nanoparticles and the amount of Se^0^ measured was equivalent to the amount of selenium originally added as selenite to the growth medium. A delay of more than 24 hours was observed between the depletion of SeO_3_^2^ and the detection of SeNPs. Nearly spherical-shaped SeNPs were mostly found in the extracellular environment whilst rarely in the cytoplasmic compartment. Size of SeNPs ranged from 50 to 400 nm in diameter, with dimensions greatly influenced by the incubation times. Different SeITE01 protein fractions were assayed for SeO_3_^2−^ reductase capability, revealing that enzymatic activity was mainly associated with the membrane fraction. Reduction of SeO_3_^2−^ was also detected in the supernatant of bacterial cultures upon NADH addition.

**Conclusions:**

The selenite reducing bacterial strain SeITE01 was attributed to the species *Bacillus mycoides* on the basis of phenotypic and molecular traits. Under aerobic conditions, the formation of SeNPs were observed both extracellularly or intracellullarly. Possible mechanisms of Se^0^ precipitation and SeNPs assembly are suggested. SeO_3_^2−^ is proposed to be enzimatically reduced to Se^0^ through redox reactions by proteins released from bacterial cells. Sulfhydryl groups on peptides excreted outside the cells may also react directly with selenite. Furthermore, membrane reductases and the intracellular synthesis of low molecular weight thiols such as bacillithiols may also play a role in SeO_3_^2−^ reduction. Formation of SeNPs seems to be the result of an Ostwald ripening mechanism.

## Background

Although selenium can be considered an essential micronutrient for living systems at low concentrations, it becomes toxic at greater doses and the range between dietary deficiency (< 40 μg day^−1^) and excess (> 400 μg day^−1^) is fairly narrow [1]. Selenium generally occurs in relatively low amounts in geological raw materials (*e.g.* native rocks and ores), soils and sediments, but its contents in coals and crude oils can reach hundreds of mg kg^−1^ in certain cases [2]. Concentrations in soils and sediments vary geographically, depending on the parent rock, ranging from 0.01 mg kg^−1^ in deficient areas to 1200 mg kg^−1^ in organic rich soils in toxic areas [3]. Therefore, selenium contamination represents an important public health concern and requires remediation initiatives especially in those geographic locations where agricultural irrigation drainage waters transport significant amounts of Se by leaching seleniferous soils. Furthermore, industrial activities such as oil refining, phosphate and metal ore mining and coal fire-based power production can all contribute to the dispersion of selenium in the environment. Se is also used extensively in both the electronics and glass industry and is added to animal feeds and food supplements. Other applications are in photocopying, in metal alloys for batteries, in vulcanized rubber manufacturing, in production of pigments, ceramics, plastics and lubricants, and in formulation of specific commodities such as anti-dandruff shampoos [4]; all of which ensure possible routes for the mobilization of selenium in the biosphere. Selenium occurs in four valence states: selenate (Se^6+^), selenite (Se^4+^), selenide (Se^2−^), and elemental selenium (Se^0^), and can form compounds with oxygen, sulfur, metals, and/or halogens [5]. The environmental fate and the toxicity of selenium strongly depend on its chemical speciation, with water soluble, oxidized forms (oxyanions) selenite (SeO_3_^2−^) and selenate (SeO_4_^2−^) showing severe toxicity to biota [5,6]. Microorganisms play a major role in the biogeochemical cycle of selenium in the environment [7]. Certain strains, that are resistant to selenium oxyanions and reduce selenite and/or selenate to the less available elemental selenium or to methylated Se forms [8], may be potentially used for the bioremediation of contaminated soils, sediments, industrial effluents, and agricultural drainage waters. It is worth noting that a large number of bacterial species, residing in diverse terrestrial and aquatic environments, possess the ability to reduce selenite and selenate into elemental selenium. This can occur through both enzymatic or non enzymatic mechanisms, leading to the formation of Se nanostructured particles (SeNPs) which are deposited inside the cell (cytoplasmic), within the periplasm or extracellularly [9-14]. Evidence exists that the microbial reduction of selenite occurs under both anaerobic and aerobic conditions. However, to date, anaerobic respiration is considered the most likely mechanism for selenite transformation to Se^0^ by means of dissimilative metabolism [15-18]. Anaerobic respiration of selenite has also been shown to involve selenite and/or selenate reductases, nitrite reductases and sulfite reductases [11,18-20]. Furthermore, the involvement of thiol-containing proteins such as glutathione has even been identified in some Gram negative bacteria capable of anaerobic reduction of Se0_3_^2−^ to amorphous Se^0^ nanoparticles [21].

These particular SeNPs display special physical characteristics such as photoelectric, semiconducting and X-ray-sensing properties [22] which make them attractive for possible nano-technological applications. They also possess adsorptive ability, antioxidant functions and due to their high surface area-to-volume ratio, a marked biological reactivity [23]; including anti-hydroxyl radical efficacy, a protective effect against DNA oxidation [24] and anti-microbial activity. Indeed, SeNPs have been found to strongly inhibit growth of *Staphylococcus aureus*, a key bacterial pathogen commonly occurring in human infections [25]. However, concern is now growing for the environmental impact of nanoparticle synthesis based on physico-chemical methods that require for high pressures and temperatures, are energy consuming, use toxic chemicals, and generate hazardous by-products. Consequently, applications using biological systems such as microbial cultures for the production of metal nanoparticles, including SeNPs, are becoming increasingly a realistic perspective. In the present paper the reduction of selenite by a strain of *Bacillus* sp. (previously classified as *Bacillus mycoides* SeITE01 [26]) has been investigated. This strain has been shown to be highly resistant to selenite (up to 25 mM) and able to transform this oxyanion into elemental SeNPs. In particular, a detailed comparison is given between the dynamics of disappearance of selenite from the growth medium and the appearance of SeNPs. Evidence is also provided for the SeNPs formation to be mainly in the extracellular environment. Based on the findings of microscopic analyses, coupled with biochemical and metabolic assays, hypotheses are advanced about possible mechanisms of reduction of selenite by *B. mycoides* SeITE01, compatible with the appearance of Se^0^ nanoparticles both inside or outside the bacterial cell.

## Results and discussion

### Taxonomic identification of the strain SeITE01

The bacterial strain SeITE01 was isolated from the rhizosphere of the Se-hyperaccumulator plant *Astragalus bisulcatus* grown on a Se-polluted soil through enrichment cultures spiked with 2.0 mM sodium selenite, as described previously [26]. It was originally hypothesized that strain SeITE01 belonged to the *Bacillus mycoides* species on the basis of partial 16S rRNA gene sequence. In the present work, a combined approach using both gene sequencing analysis and evaluation of morphological traits has provided strain SeITE01 with a definitive taxonomic position.

**Figure 1:**
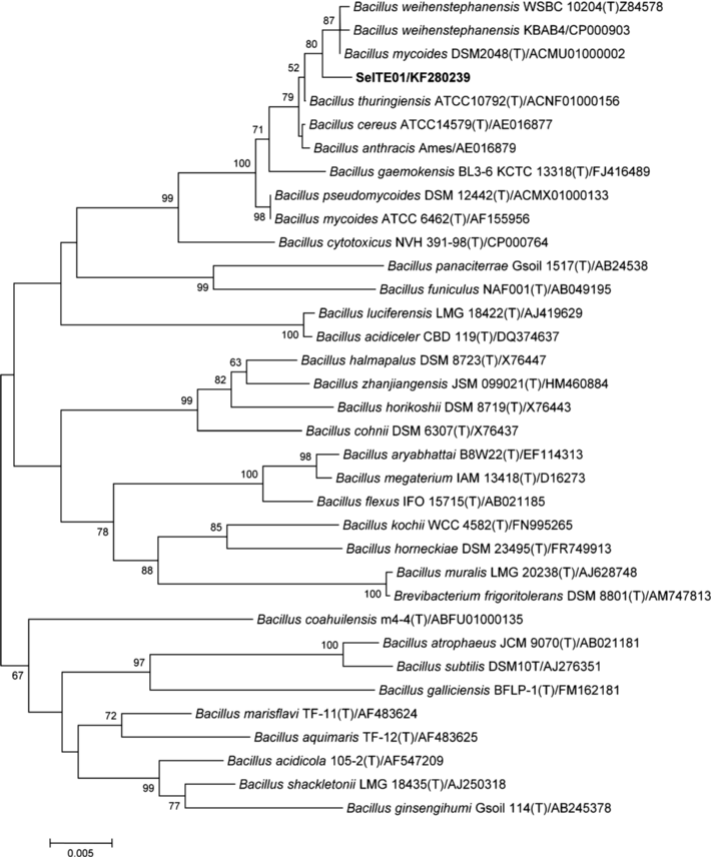
**Neighbour-joining tree inferred through MEGA 5.0 software **[61]** based on the sequences of 16S rRNA gene, showing the phylogenetic relationship of strain SeITE01 and related species.** Bootstrap values are shown for nodes that had >50% support in a bootstrap analysis of 1000 replicates. The scale bars indicate the number of substitutions per nucleotide position.

**Figure 2:**
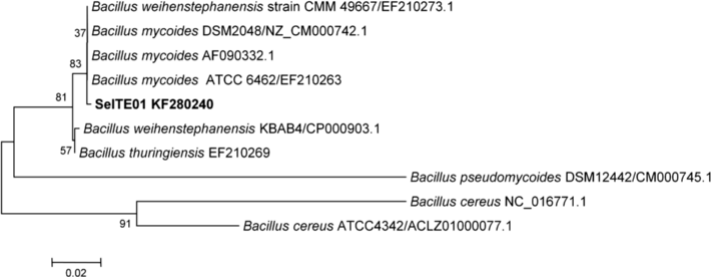
**Neighbor-joining tree inferred through MEGA 5.0 software **[61]** based on the sequences of GyrB gene, showing the phylogenetic relationship of strain SeITE01 and related species.** Bootstrap values are shown for nodes that had >50% support in a bootstrap analysis of 1000 replicates. The scale bars indicate the number of substitutions per nucleotide position.

Sequencing of the whole 16S rRNA gene confirmed that strain SeITE01 can be associated to the *Bacillus cereus* group which includes *B. cereus*, *B. thuringensis*, *B. anthracis*, *B. mycoides*, *B. pseudomycoides, B. cytotoxicus* and *B. wheihenstephanensis*[27]. Similarity values for 16S rRNA gene obtained through EZ-Taxon server [28] provided strain SeITE01 identity percentages of 99.53 and 99.40% with *B. thuringensis* and *B. mycoides* respectively; 99.31% with *B. wheihenstephanensis*; 99.27% with *B. cereus* and *B. anthracis,* and 98.58% with *B. pseudomycoides*. Neighbor-joining (N-J) phylogenetic tree showed that SeITE01 is very close to *B. mycoides* and *B. wheihenstephanensis* since they formed a separate cluster (Figure 1). These high similarity values are not surprising due to the very close relatedness among species within the *B. cereus* group which only differ from each other by zero through nine nucleotides in 16S rRNA gene sequences [29]. Thus, the mere analysis of ribosomal genes is not enough to definitively establish the attribution of the strain SeITE01 to any given species. For this reason, partial sequencing of GyrB gene [30], coding for the subunit B of the gyrase enzyme, was performed allowing the confirmation of a close connection of SeITE01 with *B. mycoides* and *B. wheihenstephanensis* on the basis of the N-J philogenetic tree (Figure 2).

**Figure 3:**
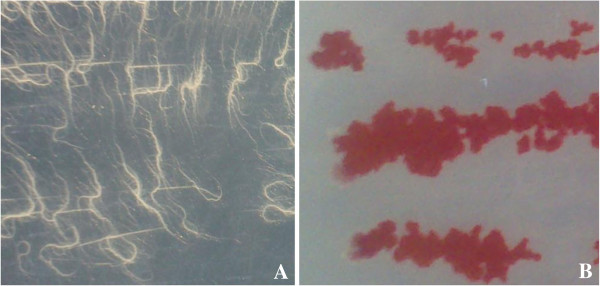
**Growth of *Bacillus *** **SeITE01 on agarized medium in absence (A) and presence (B) of 2.0 mM selenite.**

Phenotypic analysis of the bacterial growth showed that SeITE01 spreads on Nutrient agarized plates with thin, branching projections (rhizoid growth) (Figure 3). This elaborated chiral colony pattern was first described as a typical trait of *Bacillus mycoides* species by Flügge in 1886. The author called the species “mycoides” just due to fungal-like growth of these rod shaped bacteria on agar plates with filaments of chained cells projecting radially and turning left or right [31]. Interestingly, this phenotypic trait is however absent in *B. wheihenstephanensis*[32]. Thus, on the basis of such molecular and phenotypic features, the strain SeITE01 can be taxonomically positioned at the branch tip of the *B. mycoides* species.

*B. mycoides* is a common soil bacterium, occurring in the rhizosphere of different plant species. A number of studies report on the contribution of this bacterial species to the Induced Systemic Resistance (ISR) in plants even by PGP (plant growth promoting) traits [33]. In general, *Bacillus* has been recognized for its biotechnological applications at an industrial scale. Recent investigations have shown the potential of *Bacillus* species to generate biofuels (e.g. hydrogen), biopolymers (e.g. polyhydroxyalkanoates), and bioactive molecules (e.g. acyl-homoserine lactonases) [34]. Moreover, several strains of *Bacillus* sp. have been considered for bioremediation due to their degradative efficiency toward toxic organic compounds and their capacity of reducing oxyanions such as selenate and selenite to elemental selenium with formation of Se^0^ nanoparticles (SeNPs) [11,12,16,35-37].

### Testing for selenite reduction and elemental selenium formation by the strain SeITE01

**Figure 4:**
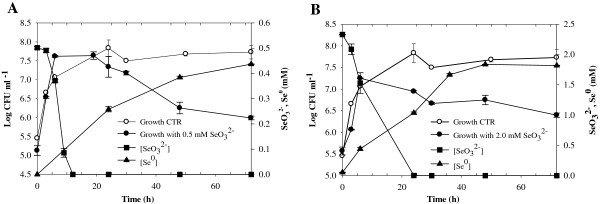
**Time courses of bacterial growth, SeO**_**3**_^**2− **^**depletion, and Se**^**0 **^**formation by *B. mycoides *SeITE01, in presence of (A) 0.5, and (B) 2.0 mM SeO**_**3**_^**2−**^**.** Each curve shows means based on the results of three experiments. Minor ticks (5-hours range) are inserted in the Time axis.

The capability of SeITE01 to transform selenite to elemental selenium was tested in liquid rich medium (Nutrient Broth) at 0.5 and 2.0 mM concentration of Na_2_SeO_3_ (Figure 4). Selenite concentration in the growth medium, elemental selenium content, and bacterial growth were all measured.

Due to the reduction of selenite, strain SeITE01 displayed a progressive depletion of the SeO_3_^2−^ initially added to the culture medium (Figure 4). Reduction of selenite was observed within 12 and 24 hours, when 0.5 and 2.0 mM SeO_3_^2−^ were supplied, respectively. At both selenite concentrations tested, the reduction process started concomitantly with the onset of the microbial growth. No lag phase was observed thus suggesting a constitutive reduction pathway. In the presence of 0.5 mM SeO_3_^2−^, the total amount of selenite initially added to the cultures was exhausted during the exponential phase of growth. By contrast, when 2.0 mM SeO_3_^2−^ was supplied, only 25% of the initial selenite content was reduced during the exponential phase, the remaining selenite being depleted during the stationary phase.

SeO_3_^2−^ negatively affected the growth dynamics of SeITE01 and final cell yield (Figure 4). At the beginning of the stationary phase no significant differences were observed on cell concentrations between selenite-supplemented cultures compared with controls. Nevertheless, the stationary phase was reached by SeITE01 more rapidly with SeO_3_^2−^ in the medium than in cultures without selenite. In particular, for cultures with no selenite added, the stationary phase was reached after about 24 hours, whereas in cultures containing SeO_3_^2−^ the stationary phase was attained after only 6–10 hours of growth. When selenium was added as 0.5 mM SeO_3_^2−^, stationary phase was prolonged up to the 20th hour with values comparable to those seen with control cultures. After 20 hours of incubation, a decrease in cell growth was observed corresponding to a reduction of about 0.1 Log units in the final cell yield with respect to control experiments. In cultures spiked with 2.0 mM SeO_3_^2−^, a decrease in cell growth was recorded just after 6 hours of incubation. These culture conditions also resulted in a lower final cell yield (1 unit Log) when compared to controls. Therefore, it seems clear that selenite exerts a toxic effect on the growth of SeITE01 with toxicity dependent on SeO_3_^2−^ concentration. These data suggested that rate and efficiency of selenite reduction are most likely related to both the initial selenite concentration and the total number of bacterial cells, rather than to the bacterial growth phase.

Reduction and consequent depletion of SeO_3_^2−^ were accompanied by the appearance of a bright red color in the growth medium. This characteristic red color was due to excitation of the surface plasmon vibrations of the monoclinic selenium (m-Se) particles [38]. Despite the reduction process running parallel to the microbial growth, red color in cell suspensions appeared later. In particular, bacterial cultures turned red after 6 and 9 hours from the start of the growth assays when supplied with 0.5, and 2.0 mM SeO_3_^2−^, respectively.

These results were in agreement with elemental selenium levels measured in bacterial cultures. In the presence of 0.5 mM SeO_3_^2−^, although the initial amount of selenite was completely depleted after 12 hours of incubation, only ~25% of it was transformed into detectable Se^0^. Moreover, after 24 and 72 hours of incubation, only ~50% and ~90% of initial selenite was respectively converted into elemental selenium. Similar results were observed when 2.0 mM selenite was added to the cultures. While the whole initial SeO_3_^2−^ content was completely reduced within 24 hours of growth, only about 50% of it was transformed into Se^0^ and about 88% of selenite resulted in the formation of Se^0^ after 72 hours. Thus, at both selenite concentrations tested, Se^0^ bioprecipitation was delayed in respect to selenite depletion in the culture medium.

This indicated that SeO_3_^2−^, before ultimate reduction to Se^0^, is likely transformed to an intermediate Se reduced form. This phenomenon has been previously observed also by Van Fleet-Stalder and co-workers [39] while studying a *Rhodobacter sphaeroides* strain capable of reducing selenite to red elemental selenium. These authors demonstrated that their bacterial strain metabolized selenite into approximately 60% RSeR and 40% Se^0^ when it was supplied with low selenite concentration (10 μM) but produced almost 100% Se^0^ after exposure to 1.0 mM selenite.

Again, Sarret and colleagues demonstrated that selenite addition into cultures of *Ralstonia metallidurans* CH34 was followed by a lag of slow uptake, during which the bacteria contained Se^0^ and alkyl selenide in equivalent proportions [40]. Subsequently, selenite uptake strongly increased and Se^0^ resulted as the predominant transformation product, suggesting an activation of selenite transport and reduction systems after several hours of contact. The authors indicated that two reactions took place in *R. metallidurans* CH34: an assimilatory pathway leading to alkyl selenide and a detoxification pathway leading to Se^0^. The identification of a SAM dependent methyltransferase (SefB) in an operon adjacent to the SeNP assembly protein SefA in *T. selenatis* has also suggested a link between both reductive and alkyl-selenide dependent selenite detoxification [14]. Moreover, Kessi and Hanselmann, while investigating the possible involvement of the Painter type reaction in selenite reduction to elemental selenium in *Rhodospirillum rubrum* and *Escherichia coli,* hypothesized at first a quick formation of a selenium-digluthathione intermediate followed by elemental selenium production [21].

### Localization of Se^0^ nanoparticles in SeITE01 cultures

**Figure 5:**
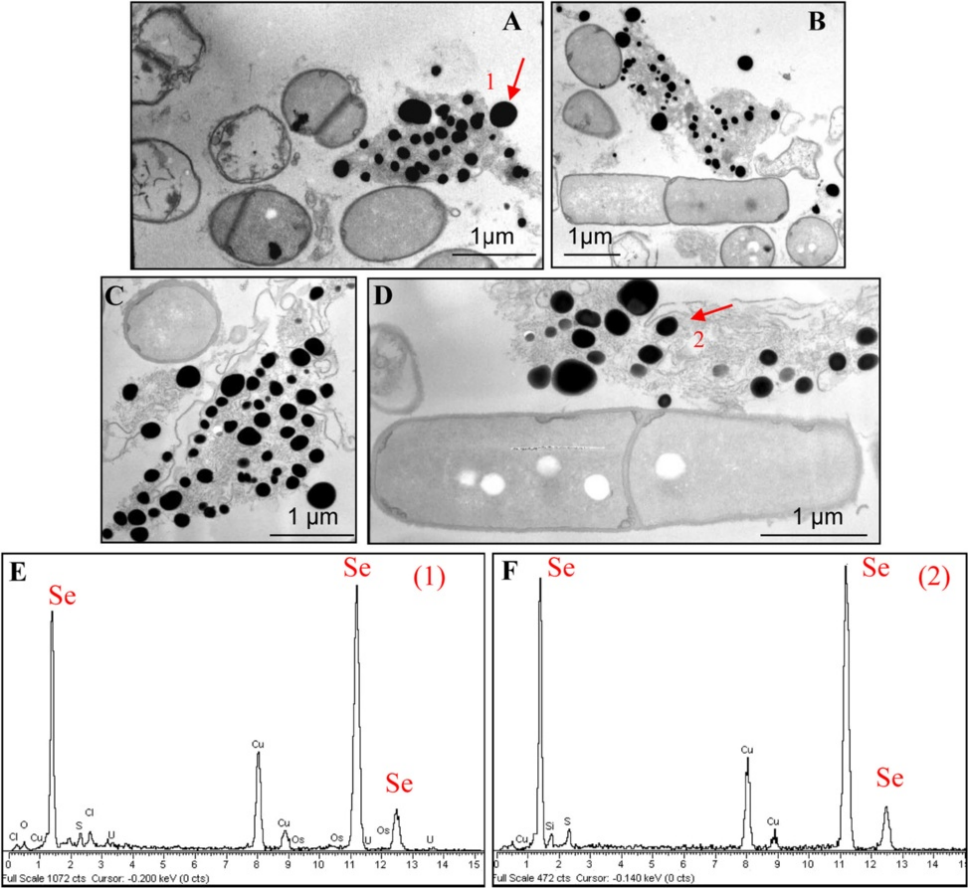
**TEM micrographs and EDAX spectra of *B. mycoides *SeITE01 cultures grown in presence of 2 mM SeO**_**3**_^**2− **^**registered at different incubation times.** 12 **(A** and **B)** and 24 **(C** and **D)** hours. Arrows point electron-dense particles (indicated by number 1 and 2), whose corresponding EDAX spectra are given on the bottom of the micrographs **(E** and **F)**.

TEM analysis (Figure 5) revealed the presence of extracellular electron-dense particles after 12 (Figure 5A) and 24 (Figure 5B) hours of SeITE01 incubation in cultures supplemented with 2.0 mM selenite. Only in very few cases, particles of the same aspect could be observed in the cytoplasm. Electron-dense granules were not detected in cell cultures which had not received SeO_3_^2−^ (data not shown). These nanoparticles seemed to be embedded in an extracellular matrix probably formed by components actively secreted or leaked out of damaged cells. However, spoiled cells or cell-like structures lacking internal organization were rarely identified in specimens examined by TEM.

EDX spectra of these nanospheres clearly indicated the presence of selenium, as the specific absorption peaks at 1.37, 11.22, and 12.49 keV were recorded (Figure 5C, D). Cu and Ni peaks could be associated with the TEM grid, whereas O and C peaks are most likely from cell components. The lack of peaks corresponding to other metals signified that selenium occurred in its elemental state (Se^0^) rather than as a metal selenide. Exposure of SeITE01 cultures to 2 mM SeO_3_^2−^ also induced formation of white granules of polyhydroxybutyrate possibly connected to stress conditions (Figure 5B and C) and caused a slight increase (1.5 time on average) of the bacterial cell length (data not shown).

**Figure 6:**
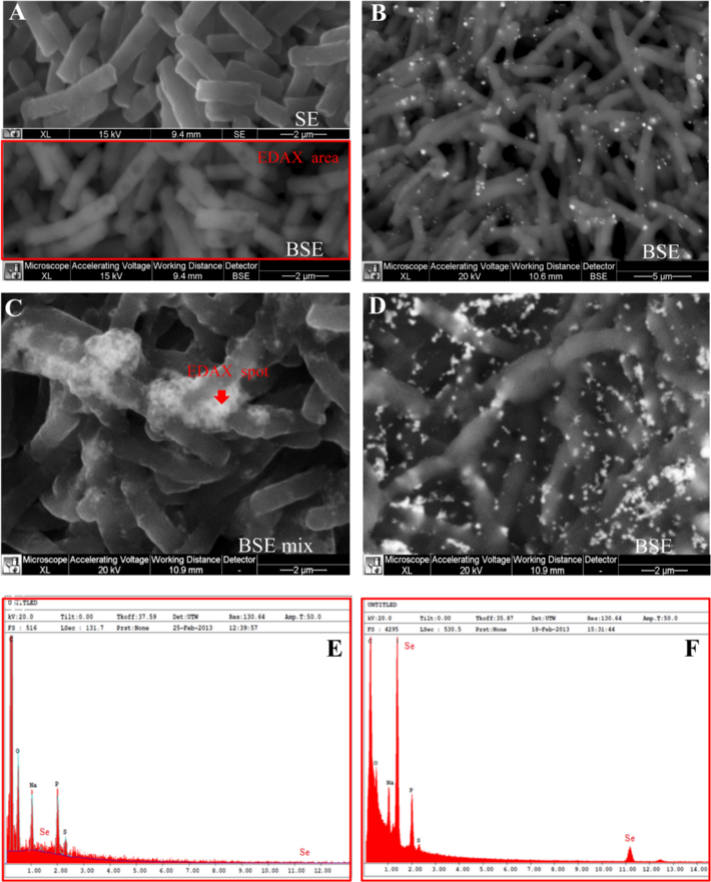
**SEM micrographs and EDAX spectra of *B. mycoides *SeITE01 cultures grown in absence (A), or in presence of 2.0 mM SeO**_**3**_^**2−**^**, at increasing incubation times: 6 (B), 24 (C), and 48 (D) hours, in panel (E) and (F) are shown EDAX spectra corresponding to control and 24 hours SeITE01 culture, respectively.** SE and BSE stand for Secondary Electrons and Back-Scattered Electrons signal, respectively.

SEM-EDX analysis carried out at different incubation times on cultures grown on 2.0 mM SeO_3_^2−^, confirmed the presence of extracellular Se^0^ nanospheres during early phases of the bacterial growth (after 6 hours of incubation) (Figure 6). Given that it is unlikely that particles of the size observed could be transported first through the plasma membrane with a vesicle-mediated excretion mechanism and then through the thick peptidoglican wall. It is therefore reasonable to infer that the reduction of selenite primarily occurs in the extracellular environment. However an ancillary mechanism of selenite reduction seems to exist, involving elemental selenium formation within the cell, either in the periplasm or in the cytoplasm, as confirmed by TEM analysis.

SeNPs appeared spherical or oblong in shape and decidedly dishomogeneous in terms of size. Both number and dimensions of these particles rose by increasing incubation time. Figure 6B shows that SeNPs possess an average diameter of 50–100 nm after 6 hours of incubation, coinciding with the exponential phase of bacterial growth, while in the late stationary phase (after 48 hours of growth) their dimensions range from 50 to 400 nm (Figures 5 and 6). This suggests that small nanoparticles, produced early in the growth phase, can behave as seeds of nucleation for further growth through a maturing process resembling the Ostwald ripening phenomenon [38].

**Figure 7:**
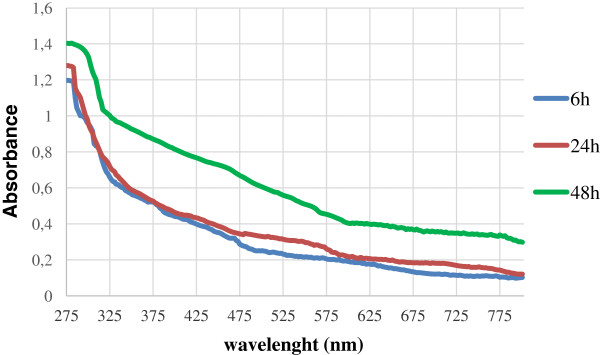
Time dependence of the UV–vis spectrum of SeNPs collected at different incubation times: (blue line) 6 h, (red line) 24 h, and (green line) 48 h.

Results in agreement with those here presented have been described for other bacterial strains able to induce the formation of Se^0^ nanoparticles by selenite reduction. *Bacillus subtilis*, *Pantoea agglomerans* UC-32 and *Shewanella* sp. HN-41 all have shown to produce SeNPs of size and shape depending on time of incubation [38,41,42]. Further characterization of the selenium nanospheres formed by strain SeITE01 was also carried out using UV-Visible absorption spectroscopy (Figure 7). SeNPs were analyzed at three different incubation times, 6, 24 and 48 hours. All spectra presented a recurrent absorption peak at 280 nm probably due to the presence of aromatic amino acids, thus indicating possible adhesion of proteinaceous material on the surface of SeNPs. These data are consistent with the previously recognized occurrence of peptides and proteins associated to SeNPs of bacterial origin [14,43,44]. In particular, Lenz and co-workers showed that selenium nanoparticles can be coupled with a variety of high-affinity proteins. For instance, they demonstrated that a protein (RarA) next to a metalloid reductase was associated with Se-nanoparticles formed by *Sulfurospirillum barnesii* SES-3 [44]. The work of Debieux et al., [14] has also identified a secreted protein (SefA) from *T. selenatis* that has been demonstrated to stabilize the formation of SeNPs during selenate respiration.

### Mechanism of SeO_3_^2−^ reduction and Se^0^ formation in *Bacillus mycoides* SeITE01

Various enzymatic systems have been proposed to explain the reduction of selenite in bacteria. In *Thauera selenatis*, the reaction might be catalyzed by a periplasmic dissimilatory nitrite reductase [45,46] or by intracellular thiols (like glutathione) [14]. In the case of *Enterobacter cloacae* reduction of SeO_3_^2−^ seems to rely on a nitrite reductase or may be carried out by intracellular glutathione [13,20]. A periplasmic reducing activity was proposed for the dissimilatory reduction of selenite by *Bacillus selenitireducens*[16]. Where enzymatic activity has been demonstrated to play a role in selenite reduction, it is mainly associated with bacterial strains capable of reducing selenium oxyanions under anaerobic conditions. In some cases, the overexpression of a single enzyme capable of reducing selenite has been ruled out. For example, in *Rhodobacter sphaeroides* the involvement of some chaperones, an elongation factor, and some enzymes associated with oxidative stress reactions was demonstrated [47]. Similar results were obtained by Antonioli and colleagues [48] through the proteomic analysis of soluble protein fractions in cells of *Stenotrophomonas maltophilia* SeITE02 grown in the presence of selenite.

Indeed, selenite can be reduced to elemental selenium by reaction with reactive thiol groups of proteins/peptides in the so called “Painter-type” reaction, which has been suggested as a general microbial detoxification reaction to oxyanions [49]. Kessi and Hanselmann [21] investigated the possible role of glutathione (GSH)/glutathione reductase (GR) system in the formation of Se^0^ nanoparticles from SeO_3_^2−^. In their experiments using the phototrophic proteobacterium *Rhodospirillum rubrum,* these authors showed that the rate of selenite reduction declined when bacteria were synthesizing lower than normal levels of glutathione, while in *Rhodobacter sphaeroides* and *Escherichia coli* SeO_3_^2−^ reduction was reported to induce glutathione reductase activity. Garbisu and co-workers also observed a significant induction of thioredoxin and thioredoxin reductase in *Bacillus subtilis* exposed to millimolar concentrations of selenite [35]. This detoxification mechanism was further supported in the study by Lenz and co-workers [44], since peroxiredoxins which contain catalytic cysteine-thiols were identified in *B. selenatarsenatis*.

**Figure 8:**
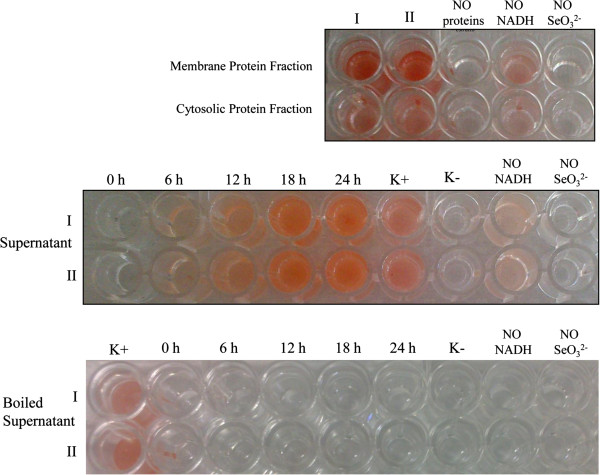
**Selenite reduction assay carried out on protein fractions (shown at the top) and on supernatant, boiled and not boiled, (shown at the bottom) of SeITE01 liquid cultures.** All tests were done in duplicate (indicated by roman numbers), with addition of 2.0 mM SeO_3_^2−^ and 2.0 mM NADH. Three negative controls were set up: without protein fractions or supernatant, without selenite, without NADH.

To clarify the mechanism of selenite reduction to elemental selenium in *B. mycoides* SeITE01, a number of SeO_3_^2−^-reduction assays were carried out. On the basis of electron microscopic analyses, which suggested that SeNPs formation was occurring outside the cell, cell protein fractions (i.e. cytosolic and membrane-associated) and supernatant from liquid cultures were analyzed for the presence of selenite reducing activity. Moreover, to define a possible role of exopolysaccharides (EPS) in the formation of SeNPs, a selenite reduction assay was also performed on the EPS fraction extracted from SeITE01 bacterial cultures. As shown in Figure 8, selenite reduction occurred mainly in the fraction of membrane-associated proteins after the addition of NADH, with only very little activity detected in the cytosolic fraction. By contrast, no reduction activity was found in the EPS fraction (data not shown). SeO_3_^2−^ reduction was also observed in the supernatant of SeITE01 cultures, although again only after NADH addition. Boiling the supernatant samples resulted in a complete loss of reduction activity, inferring an enzymatic rather than chemical catalyzed reaction. Therefore, based upon the combined evidence two different mechanisms could account for the reduction of selenite into SeNPs in *Bacillus mycoides* SeITE01. The main mechanism is proposed to involve the action of proteins/peptides, released by bacterial cells or activated at the plasma membrane or wall surface. These proteins/peptides may function as oxido-reductase enzymes or proton antitransporters. SeO_3_^2−^ would be reduced to form Se^0^ seeds by interacting with these proteins. Sequentially, Se^0^ seeds would grow into large SeNPs by further reduction of SeO_3_^2−^ and aggregation of Se atoms through an Ostwald ripening mechanism [24]. Meanwhile, an ancillary mechanism consisting of the intracellular reduction of selenite and involving enzymatic membrane activity may exist. In this case, SeNPs would grow inside the cell and then leak out into the extracellular space after cell lysis.

Data recorded on selenite depletion in SeITE01 cultures clearly demonstrate that the reduction of SeO_3_^2−^ occurs well before the appearance of Se^0^ nanoparticles. This might provide further support for the hypothesis that the formation mechanism of SeNPs is actually a two-step reaction. Selenite is possibly first rapidly reduced by thiol groups occurring in extracellular peptides or proteins resulting in the formation of selenides. These could then be hydrolyzed releasing nanometer-sized particles of elemental selenium which undergo extracellular precipitation.

Recently, Dwivedj and co-workers showed that *Pseudomonas aeruginosa* JS-11 was capable of synthesizing SeNPs trough extracellular reduction of selenite [50]. As for the strain SeITE01, a selenite reduction activity was observed in the spent medium of *P. aeruginosa* JS-11 and the involvement of NADH and NADPH dependent reductases as well as of the metabolite phenazine −1-carboxylic acid (PCA) released by strain JS-11 in the supernatant has been suggested. It is finally worth noting that a chromate reductase has been described as secreted enzymatic protein in *Bacillus amyloliquefaciens*[51]. In this case, the microbial cells do relay on a specific extracellular mechanism to face metal toxicity. Even more lately, a protein showing NADH-dependent reductase activity capable of converting SeO_3_^2−^ to Se^0^ has been described in cell-free extracts of *Rhizobium selenitireducens*[52].

However, additional experiments are needed to better understand both the nature and the release mechanism of such extracellular reductases in *B. mycoides* SeITE01.

## Conclusions

**Figure 9:**
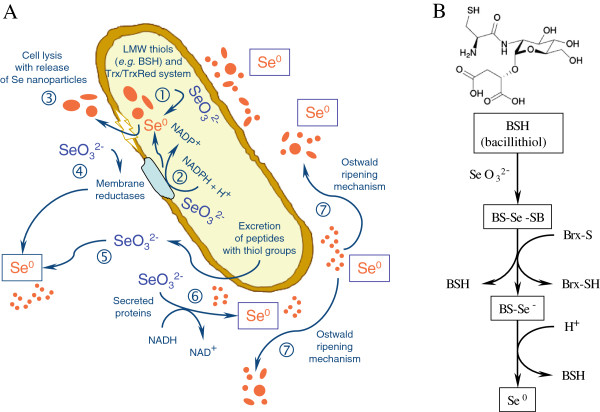
**Hypothesis of SeNPs formation in *Bacillus mycoides *SeITE01. A** - Synoptic schematization of proposed biogenesis mechanisms of zero-valent selenium nanoparticles in *Bacillus mycoides* SeITE01. (1) Cytosolic precipitation of SeO_3_^2−^ as Se^0^ nanoparticles due either to the possible activity of LMW thiols including bacillithiol (BSH) or to the Trx/TrxRed system. (2) Intracellular selenite reduction and formation of SeNPs as a consequence of presumable activity of membrane reductases. (3) Release of intracellularly generated SeNPs after cell lysis. (4) Membrane reductases may even catalyze extracellular selenite precipitation. (5) Peptides and other compounds carrying thiol groups may be released from the bacterial cell and directly react with selenite. (6) Evidence of the formation of SeNPs by *Bacillus mycoides* SeITE01 culture supernatant spiked with SeO_3_^2−^ only after NADH addition can be due to the presence of extracellular proteins capable of mediating selenite precipitation once provided with reducing equivalents. (7) Nascent SeNPs are inherently unstable due to their high surface area and therefore tend to grow and increase their average size to attain a lower-energy state by means of an Ostwald ripening mechanism. **B** - Suggested mechanism of selenite detoxification in *Bacillus* sp. involving Brx-like proteins, according to [55].

In conclusion, the bacterial strain SeITE01 isolated from the rhizosphere of the selenium hyperaccumulator legume *Astragalus bisulcatus* grown in a Se contaminated soil has been taxonomically attributed to the species *Bacillus mycoides* on the basis of phenotypic and molecular traits. It has the ability to induce the formation of amorphous Se^0^ nanoparticles under aerobic conditions as a consequence of the reduction of selenite. Not only extracellular but also intracellular elemental selenium production was detected, although accumulation of SeNPs was mostly observed outside the bacterial cell. The size of SeNPs was dependent on the incubation times, showing a direct relationship between incubation time and the nanoparticle size. Increasing the incubation time increases the size of SeNPs observed. Based on the results, a tentative explanation for the process of SeNPs formation can be given (Figure 9). It is proposed that SeO_3_^2−^ ions are reduced into Se^0^ by the concourse of enzymatic proteins released by the bacterium and may also react directly with sulfhydryl groups on thiols of peptides released by *Bacillus* cells. Furthermore, membrane reductases may play a role in SeO_3_^2−^ reduction. Selenite ions once reduced form Se nuclei which, subsequently, grow into the large SeNPs by further reduction of SeO_3_^2−^ ions and an aggregation of these Se atoms, involving an Ostwald ripening mechanism [24]. Small SeNPs are then consumed for the growth of larger ones according to the Gibbs–Thomson Law [53]. As mentioned previously, SeITE01 cultures grown in the presence of selenite demonstrated the presence – although sporadic – of spherical intracellular deposits of SeNPs by TEM analysis. In this regard, bacillithiol (BSH) has been identified as a major low-molecular-weight (LMW) thiol playing a significant role in the cytosolic thiol redox chemistry of low G + C Gram-positive bacteria such *Bacillus* sp., concomitantly with the functions of other LMW thiols (e.g. cysteine residues) or Trx/TrxRed pathways [54]. BSH-synthesizing bacteria may contain enzymes analogous to those found in GSH-containing bacterial species, with bacilliredoxin (Brx) instead of glutaredoxin (Grx). Although the reductase system capable of maintaining BSH in the reduced state is not fully understood so far in *Bacillus* sp., the involvement of Brx-like proteins in a pathway analogous to that observed with GSH in Gram-negative bacteria may therefore be claimed in the strain SeITE01 for a complementary detoxification of selenite through reduction to Se^0^ with later intracellular precipitation in form of SeNPs [55,56]. Finally, although the formation of a selenium intermediate is only presumptive in this study, it has been previously suggested [14,21,39,40] as discussed above. Therefore, additional studies have to be made to identify the possible intermediates in *Bacillus mycoides* SeITE01.

## Methods

### Chemicals, culture media and solutions

Chemicals purchased from Sigma-Aldrich (Milan, Italy) were all analytical grade. Nutrient Broth, and Bacteriological Agar were furnished by Oxoid Italia Spa (Garbagnate Milanese, Italy). Na_2_SeO_3_ was prepared as a 100 mM stock solution in deionized water and sterilized by filtration.

### Bacterial strain SeITE01 and culture conditions

Bacterial strain SeITE01 was obtained by means of enrichment cultures supplied with selenite 2.0 mM from the rhizosphere of the selenium hyperaccumulator plant *Astragalus bisulcatus*, grown on a Se-polluted soil [26]. After isolation, the strain was maintained in Nutrient medium added with 2.0 mM selenite. Storage was in 30% glycerol at −80°C.

### Taxonomical analyses

Total DNA was isolated from 18-h bacterial cultures grown on Nutrient medium by using the NucleoSpin Tissue Kit (Clontech) according to manufacturer’s instructions. 16S rRNA and GyrB genes were amplified through PCR using respectively F8/R11 [57] and BMSH-F/BMSH-R [30] primer sets. Conditions for 16S rRNA gene amplification were as follow: 95°C for 5 min, then 30 cycles of 95°C for 1 min, 50°C for 1 min, 72°C for 2 min, with a final extension step at 72°C for 5 min. The PCR program for GyrB gene amplification was as reported in [30].

PCR products were cloned into pGEM-T vector through the Easy T-Vector System (Promega, Italy), following the manufacturer’s instructions and then sequenced on both strands (Primm, Italy). Identification of phylogenetic neighbors for 16S rRNA gene sequence was initially carried out by BLAST [58] and megaBLAST [59] programs against the database of type strains with validly published prokaryotic names [28]. The fifty sequences with the highest scores were then selected for the calculation of pairwise sequence similarity using global alignment algorithm, which was implemented at the EzTaxon server (http://eztaxon-e.ezbiocloud.net/ezt_identify; [28]). GyrB sequence was searched for similarity through megaBLAST [60] relying on the NCBI database. The 16S rRNA and GyrB gene sequences were registered as accession KF280239 and KF280240 in the GenBank database.

Multiple nucleotide sequences alignments were constructed using CLUSTAL_W 1.83 [60]. Phylogenetic trees were obtained using neighbor-joining algorithms within MEGA version 5.0 software package [61] with 1000 data sets examined by bootstrapping. Missing nucleotides at both the beginning and the end of the sequences were deleted before construction of the trees.

### Evaluation of reduction efficiency by strain SeITE01 at increasing SeO_3_^2−^ concentrations

Efficiency of selenite reduction was determined for SeITE01 in rich growth medium (Nutrient Broth). All microbiological tests were carried out in 250-ml Erlenmeyer flasks containing 100 ml of growth medium incubated at 28°C on an orbital shaker (200 rpm). Each flask was inoculated with aliquots from stationary-phase cultures of the strain SeITE01 to reach a final optical density of 0.01. Assays were performed in the presence of two different Na_2_SeO_3_ concentrations, namely 0.5 or 2.0 mM. Culture samples collected at different times during different tests were analyzed for bacterial growth, residual selenite in the medium, and formation of elemental Se.

#### Microbial growth estimation

Bacterial growth was evaluated by counting the colony forming units (CFU) on agarised Nutrient Broth plates seeded with aliquots of bacterial cultures. All analyses were performed in triplicate. Bacterial growth in presence of SeO_3_^2−^ was checked vs. control cultures incubated in Nutrient Broth with no Na_2_SeO_3_ added.

#### SeO_3_^2−^ content determination

SeO_3_^2−^ concentration in culture medium was measured spectrophotometrically by using the method described by [10]. This method was carried out as follow: first 10 ml of 0.1 M HCl, 0.5 ml of 0.1 M EDTA, 0.5 ml of 0.1 M NaF, and 0.5 ml of 0.1 M of disodium oxalate were mixed in a 50 ml glass bottle. A 50- to 250-μl sample containing 100 to 200 nmol of selenite was added, and then 2.5 ml of 0.1% 2,3-diaminonaphthalene in 0.1 M HCl was amended. The bottles were incubated at 40°C for 40 min and then cooled to room temperature. The selenium-2,3-diaminonaphthalene complex was extracted with 6 ml of cyclohexane by shaking the bottles vigorously for about 1 min. The absorbance at 377 nm of the organic phase was determined by using a spectrophotometer Heλios β, Unicam. Sterile cultures were also tested for SeO_3_^2−^ concentration as negative controls. All manipulations were done in the dark.

Calibration curves were performed by using 0, 50, 100, 150 and 200 nmol of selenite in Nutrient broth.

#### Se^0^ content determination

Se^0^ concentration was measured spectrophotometrically by using the method described in [62]. A standard for elemental selenium was prepared by reducing selenite to amorphous red Se^0^ as follows: aliquots of a 0.1 M sodium selenite solution were placed in test tubes to give a range of 1 to 10 μmol selenite per tube. 25 μmol of HN_2_OH · HCl (Sigma-Aldrich) were then added to each tube containing selenite. This concentration of hydroxylamine ensured quantitative reduction of SeO_3_^2−^ to Se^0^. The tubes were gently mixed and after 1 hour, the intensity of the red-brown selenium solution was measured at 490 nm. To establish the Se^0^ standard, average values of triplicate samples were used. In order to determine the amount of selenium produced by SeITE01 strain, the bacterial culture along with the insoluble red elemental selenium was gently mixed and 10 ml was transferred to polycarbonate centrifuge tubes. After centrifugation at 5000 × g, bacterial cells and elemental selenium were collected as a pellet. Cells disruption was achieved by means of a sonicator equipped with a steel tip (Hielscher UP50H), by repeating 7 sonication cycles (40 seconds sonication alternated with 40 seconds of rest in ice), while keeping the samples always in ice. Once sonicated, pellets were washed twice with 10 ml of 1 M NaCl to remove non-metabolized selenite. The red colloidal selenium in the pellet was dissolved in 10 ml of 1 M Na_2_S and after centrifugation to remove bacterial cells, absorption of the red-brown solution was measured at 490 nm.

### Electron microscopy analysis

Cell size and shape were identified through transmission electron microscopy (TEM) or scanning electron microscopy (SEM) starting from samples of bacterial cultures grown either in Nutrient Broth or in Nutrient Broth supplied with 2.0 mM Na_2_SeO_3_, respectively.

#### TEM analyses

To obtain thin sections for electron microscopy analysis, bacterial cells were embedded in Epon-araldite resin after fixation with 2.5% paraformaldehyde + 2.5% glutaraldehyde in cacodylate buffer (0.1 M cacodylate, pH 7.2) and post-fixation with 1% OsO_4_ + 0.15% ruthenium red in cacodylate buffer as previously reported in [63]. Sections were prepared by means of a Reichert Ultracut S ultramicrotome (Leica) equipped with a diamond knife. Uranyl acetate and lead citrate were used as contrast agents.

#### SEM analyses

Bacterial cells analyzed through scanning electron microscopy underwent the same fixation and post-fixation procedure as it has been described for TEM preparations. Once fixed, cells were dehydrated with increasing ethanol concentrations and dried through the critical point method by using liquid CO_2_. Cells were mounted on metallic specimens stubs and sputter-coated with carbon (MED 010 Balzers) then directly observed through the electron microscope.

TEM observations were carried out with a high resolution electron microscope Jeol JSM 5200. Whereas Energy-dispersive X-ray (EDX) analyses were performed with a high resolution electron microscope (JEOL JEM 2010) operated at high accelerating voltage (200 kV) and equipped with an Inca 100 Link analysis system. SEM observations has been done using mainly the back-scattered electron (BSE) emission mode with XL30 ESEM (FEI-Philips) equipped with an EDAX micro-analytical system.

### Analysis of Se nanoparticles (SeNPs)

#### Recovery of selenium nanoparticles from the culturing medium

Experiments were done using 250 ml flasks, each containing 100 ml of Nutrient Broth with a selenite concentration of 2 mM. After 24 and 48 hours of growth, the culture broth was centrifuged at 10020 × *g* at 4°C for 10 min. The pellet was discarded and the cell-free medium was centrifuged at 41410 × *g* at 4°C for 30 min. The supernatant was discarded and the pellet with the selenium-containing particles was re-suspended in water. The suspension was washed twice by repeating the two centrifugation steps.

#### UV–visible spectral analysis

Absorbance was measured using double beam UV–Vis spectrophotometer at wavelengths between 275 to 800 nm. The SeNPs dispersed in deionised Milli-*Q* water were stored at room temperature.

#### Selenite reduction activity assays

To check the selenite reduction activity by SeITE01 bacterial cultures reduction activity assays were carried out starting from different cell protein components (i.e. cytosolic and membrane-associated) as well as exopolysaccharide (EPS) fraction, and supernatant of SeITE01 liquid culture.

#### Protein extraction

SeITE01 cultures were grown up to log phase (18 hours of incubation) and centrifuged at 39100 × g (Hermle centrifuge, Z36HK) for 10 min at 4°C to obtain the cell pellet. Pellet was washed twice with 10 mM Tris-Cl (pH 7.5) and re-suspended in the same buffer for sonication. After sonication, the cell lysate was centrifuged at 22540 × g for 40 min to separate the soluble and membrane fractions. Total protein content was estimated by Bradford method using BSA as standard.

#### EPS extraction

A modification of the protocol developed by [64] was adopted. After 5 days of growth, bacterial cultures were centrifuged at 12000 × g for 30 min at 4°C. Then, the supernatants were collected, filtered through a 0.45 μm membrane and precipitated overnight with three volume of cold ethanol at −20°C. The precipitated polysaccharides were centrifuged at 10000 rpm for 30 min at 4°C and resuspended in distilled water.

#### Supernatant preparation

Supernatant of SeITE01 cultures in Nutrient Broth medium was collected at different times during the bacterial growth curve, namely after 0, 6, 12, 18 and 24 hours of incubation. Samples to be analyzed were recovered by an initial centrifugation at 5000 × g followed by a filtration with 0.2 μm disks. Heat treated samples were obtained after boiling at 121°C for 15 minutes.

#### SeO_3_^2−^ reducing activity assay

The activity assay to check selenite reduction was performed as follow: 100 μL of proteins (2 mg mL^−1^) or EPS (2 mg mL^−1^) or supernatant samples were collected in 0.2 mL tubes and then carefully transferred in a 96-well microtitre plate. Subsequently, 88 μL of McIlvaine buffer, 10 μL of Na_2_SeO_3_^2−^ solution (final concentration 5.0 mM) and 2 μL of NADH (final concentration 2.0 mM) was added to each well. The mixture was then incubated at room temperature for 24 hours. Formation of red colour in the wells, indicating the production of elemental selenium, was interpreted as positive result.

## Competing interests

The authors declare that they have no competing interest.

## Authors’ contributions

SL carried out the whole taxonomic characterization of the strain SeITE01, performed TEM analyses, followed all selenite transformation tests, and drafted the manuscript. EZ focused on elemental selenium measurements, SeNPs UV-vis spectra, and selenite reduction assay on different protein fractions. CB participated in the revision of the manuscript. PB managed SEM-EDX analyses. CSB improved the manuscript text, also giving important suggestions for a better presentation of the results. GV, coordinator of the Research Unit of Microbial Biotechnology and Environmental Microbiology at the Department of Biotechnology – University of Verona, revised the whole manuscript and elaborated the hypothesis for selenite reduction mechanisms and elemental selenium formation in the strain SeITE01, as highlighted in Figure 9. All authors read and approved the final manuscript.
